# A novel tissue-engineered stent graft combining decellularized scaffold and bioresorbable stent: a pilot feasibility study in a porcine model

**DOI:** 10.1080/15476278.2025.2610591

**Published:** 2025-12-31

**Authors:** Tatsuya Shimogawara, Kentaro Matsubara, Kazuki Tajima, Masayuki Shimoda, Hiroshi Yagi, Hideaki Obara, Yuko Kitagawa

**Affiliations:** a Department of Vascular Surgery, Saiseikai Yokohamashi Tobu Hospital, Kanagawa, Japan; b Department of Surgery, Keio University School of Medicine, Tokyo, Japan; c Department of Small Animal Internal Medicine, Kitasato University School of Veterinary Medicine, Aomori, Japan; d Department of Pathology, The Jikei University School of Medicine, Tokyo, Japan

**Keywords:** Bioresorbable stent, decellularization, porcine model, tissue-engineered stent graft

## Abstract

Endovascular aneurysm repair (EVAR) is a widely accepted treatment for aortic pathologies owing to its minimally invasive nature. However, long-term complications, such as stent graft migration and infection, remain unresolved, primarily due to the persistent presence of synthetic materials and limited tissue integration. This pilot study evaluated the feasibility of a novel tissue-engineered stent graft (TESG) combining a bioresorbable poly-L-lactic acid (PLLA) stent with decellularized porcine veins. The veins were processed using a sodium dodecyl sulfate and the Triton X-100 decellularization protocol. Histological and ultrastructural analyses confirmed effective cell removal while preserving extracellular matrix components. Quantitative deoxyribonucleic acid (DNA) analysis showed a > 97% reduction in DNA content. The TESGs were assembled by suturing the decellularized veins into bioresorbable PLLA stents and implanted into porcine iliac arteries (*n* = 3). Commercially available prosthetic grafts were used as control implants to evaluate differences in tissue responses. Graft patency and morphology were assessed at implantation and on postoperative day 14 using angiography and intravascular ultrasonography. All TESGs remained patent, with no evidence of thrombosis or aneurysmal changes. Histological analysis revealed early endothelialization and smooth muscle cell infiltration within the TESG wall, in contrast to the prosthetic graft controls, which lacked comparable cellular integration. This study demonstrated the short-term feasibility and biological compatibility of a fully bioresorbable TESG. Although long-term outcomes remain to be established, these results support further development of TESG to reduce late complications through improved tissue integration and avoidance of permanent synthetic materials.

## Background

Many aortic aneurysms are treated with endovascular aneurysm repair (EVAR) using a stent graft (SG). EVAR has marked a major change in the treatment strategy for aortic aneurysms. Six SG systems are commercially available in Japan. These devices consist of impermeable prosthetic grafts (PGs) composed of polyester or expanded polytetrafluoroethylene (ePTFE) supported by either endoskeletal or exoskeletal metallic stents made from nitinol or cobalt–chromium alloys. The most significant reason for the widespread use of EVAR in the past decade is its minimal invasiveness, and worldwide evidence of its effectiveness has been established.^1–3^

However, SG has problems with possible migration and residual foreign materials. The incidence of re-intervention after EVAR has been reported approximately 20–30% in a Japanese nationwide prospective cohort study.^4^ A meta-analysis of 22 retrospective studies found a migration rate of approximately 8.6%, with approximately 39% of those cases requiring re-intervention, typically identified within 12–36 months after EVAR.^5^ Due to their limited biocompatibility, currently available commercial devices depend primarily on mechanical fixation. Moreover, the long-term persistence of synthetic materials in the body may increase the risk of late-onset infections, particularly in immunocompromised or older patients, highlighting the need for grafts with improved biological integration and resorbability.^6^

Decellularization is a tissue-engineering technology that has been adapted in clinical practice.^7–11^ Decellularized tissues preserve the original structure of the extracellular matrix (ECM) and provide an optimal environment for cellular differentiation, proliferation, and function.^12^ As demonstrated in clinical reports, decellularized allografts reduce antigenicity, thereby lowering the risk of graft dysfunction or stenosis after implantation for long periods.^13^ Early clinical trials of decellularized vessels have shown promising results.^14^^,^^15^

The Igaki–Tamai stent (ITS; Kyoto Medical Planning Co., Ltd., Kyoto, Japan) is the first bioresorbable stent (BRS) for human use produced in Japan. It is commercially available in Europe for the treatment of coronary and peripheral artery diseases and is gaining wide acceptance. The Remedy stent (Kyoto Medical Planning Co., Ltd., Kyoto, Japan) is a bioresorbable peripheral vascular scaffold developed based on the foundational technology of the ITS. The most remarkable advantage of this stent over its metallic counterparts is that its scaffold, composed of poly-L-lactic acid (PLLA), undergoes complete hydrolytic degradation into carbon dioxide and water within approximately 12-18 months.^16^ This property offers a favorable environment for subsequent revascularization procedures.

Therefore, combining these two technologies has the potential to construct a novel tissue-engineered SG (TESG). Moreover, TESG composed of biodegradable components designed to achieve biological fixation through integration with native tissues and to degrade without residual permanent foreign materials, may reduce the incidence of graft-related complications over time. Previous studies have examined TESGs incorporating partially bioresorbable synthetic materials, such as degradable polyester meshes combined with nondegradable scaffolds, which showed limited but promising remodeling potential.^17^^,^^18^ However, to the best of our knowledge, no previous approach has realized a fully bioresorbable TESG concept, making the present strategy of combining a decellularized scaffold with a bioresorbable stent an entirely novel development. The aim of the present study was to produce a bioresorbable SG using a decellularized scaffold and BRS, and to confirm its short-term feasibility and biological compatibility in porcine models.

## Materials and methods

### Animals

Three juvenile, pathogen-free, female pigs (30–40 kg; JA Zennoh, Tokyo, Japan) were used for TESG implantation. The animals were sedated with medetomidine (0.1 mg/kg) and midazolam (0.2 mg/kg), followed by inhalation anesthesia using isoflurane. Each animal received prophylactic antibiotics and perioperative antiplatelet therapy. All procedures were approved by the Institutional Animal Care and Use Committee of the Keio University School of Medicine.

### Decellularization procedure

The decellularized vein was selected as the graft-covering material because of its preserved mechanical strength, comparable to that of both native and decellularized arteries, as preliminarily demonstrated by tensile testing. Furthermore, in TESG fabrication, its thinner wall structure and superior handling characteristics were considered advantages for this application.

Porcine jugular veins were harvested from juvenile pigs (body weight: 20–30 kg) under general anesthesia. Explanted venous segments were immediately stored in cold phosphate-buffered saline (PBS) and transported to the laboratory on ice. Samples were rinsed with sterile PBS in 4 °C to remove residual blood clots. Excess connective and adventitial tissues were carefully trimmed using a scalpel. All prepared venous specimens were then preserved in sterile PBS solution and frozen at -80 °C until decellularization for at least 24 hours.

The method utilized to decellularize the porcine vein was adapted and modified from a previously reported protocol.^19^ Samples were washed once with PBS, then perfused with 0.1% sodium dodecyl sulfate solution in deionized water (DW) for 12 hours at 37 °C. They were washed again with PBS solution and perfused with 1% Triton-X solution in DW for 0.5 hour at 37 °C. Samples were finally rinsed twice with PBS for 15 minutes to remove any residual detergents and stored overnight in DW with 1% penicillin/streptomycin at 4 °C.

### Assessment of decellularization efficacy

The efficacy of the decellularization process was assessed using histological, ultrastructural, and quantitative analyzes.

#### Histological and ultrastructural evaluation

Untreated and decellularized porcine jugular veins were fixed in 4% paraformaldehyde, embedded in paraffin, and sectioned at a thickness of 5µm. Hematoxylin and eosin (H&E) staining was performed to assess general vascular morphology, and Elastica Van Gieson (EVG) staining was used to evaluate the ECM components. The surface ultrastructure of the porcine veins was analyzed using scanning electron microscopy (SEM). Samples were cut into small rectangular shapes and fixed in 2.5% glutaraldehyde solution for 2 hours, then dehydrated, freeze-dried (VFD-21S, Vacuum Device, Ibaraki, Japan) and osmium-coated to obtain nonconductive characteristics. Each sample was analyzed using a SU6600 scanning electron microscope (Hitachi, Tokyo, Japan).

#### DNA quantification

Residual DNA content in each sample (*n* = 6) was determined to evaluate the efficacy of decellularization. DNA was isolated using phenol/chloroform extraction, precipitated with ethanol, and quantified by spectrophotometry.

#### Quantitative analysis of DAPI-positive areas

4’,6-diamidino-2-phenylindole (DAPI) staining were performed to evaluate the removal of cellular nuclei. Microscopic quantification of residual nuclear material was conducted using ImageJ software (NIH, Bethesda, MD, USA). Four randomly selected microscopic fields were analyzed in both native and decellularized vascular tissues. Each image was converted to 8-bit format, and an identical threshold was applied across all images. The “Analyze Particles” function was then used to quantify DAPI-positive areas, allowing objective comparison of nuclear material between untreated and decellularized samples.

### Tensile testing

Mechanical properties of untreated and decellularized scaffolds (*n* = 6, each) were analyzed using a tensile testing device (TENSILON; Orientec Co., Tokyo, Japan) at a room temperature of 20 °C. Porcine inferior vena cava were obtained from a local slaughterhouse (Tokyo Shibaura Organ Co., Tokyo, Japan), washed with saline, and the additional soft tissues were trimmed. Decellularized scaffolds were prepared using a previously described protocol 1 day before testing. All samples were maintained in saline at 4 °C. Prior to tensile testing, 5 × 30 mm longitudinal sections were obtained from each sample using scissors. The thickness of each sample was measured using a micrometer. The samples were then mounted on a testing machine and elongated to failure strain at a rate of 10 mm/min using a 50 *N* load cell. The ultimate tensile strength was calculated from the stress–strain curve.

### Construction of TESG

A frozen porcine jugular vein of6 mm diameter was thawed overnight at 4 °C prior to decellularization. The thawed vein was decellularized 1 day before surgical implantation, according to a previously described protocol. The resulting scaffold was attached inside a 5 × 37 mm BRS with a 6-0 absorbable suture under clean conditions. The constructed TESG was sorted overnight in 1% penicillin/streptomycin antibiotic solution at 4 °C until surgical implantation.

### Surgical implantation

Surgical graft implantation was performed in an animal model to evaluate the biocompatibility of the TESG. A total of three pigs were administered general anesthesia with isoflurane. In the supine position, the bilateral external iliac and common femoral arteries were exposed and encircled using inguinal and median incisions. Prior to angiography, systemic heparinization was established with an intravenous injection of heparin (200 U/kg); heparin (1000 U) was additionally administered every 1 hour. The initial anatomical features were detected using aortic digital subtraction angiography (DSA) with an arterial retrograde contrast injection from a 4-Fr introducer sheath (Medi-kit; Tokyo, Japan) placed in both common femoral arteries. An intravascular ultrasound system (IVUS; Volcano s5 Imaging System, Volcano Corp., Rancho Cordova, United States) and a catheter (Visions PV; Volcano Corp.) were used to evaluate the luminal characteristics. The IVUS catheter was automatically retracted by a rate of 1.0 mm/s. To prevent vessel spasms, nitroglycerin (0.2 mg) was locally injected through a catheter before IVUS examination. The bilateral iliac arteries were clamped, resected to a length of 10 mm, and anastomosed with grafts.

Graft implantation was performed in an end-to-end manner using a 6-0 non-absorbable suture (PROLENE; Ethicon Inc., Somerville, United States) for TESG and a GoreTex CV-7 (Gore WL Gore and Associates, Flagstaff, United States) for ePTFE. The TESG was anastomosed to the right iliac artery and ePTFE to the left side in each animal (*n* = 3). The ePTFE graft served as a control to evaluate comparative biological response and integration. To confirm the accuracy of the anastomotic procedure, aortic angiography and IVUS were repeatedly performed. Finally, the incisions were closed with 3-0 absorbable sutures and the animals were recovered from anesthesia.

Prophylactic oral antibiotics were administered for 1 week postoperatively. Dual antiplatelet therapy (aspirin and ticlopidine) was initiated 2 days before surgery and maintained throughout the study in all animals. Thus, both the TESG and prosthetic graft groups received identical antiplatelet coverage to minimize potential thrombotic bias between the groups.

### Graft explantation

All grafts were harvested on postoperative day 14. The animals were anesthetized as described above and the left carotid artery was exposed to place a 4-Fr short sheath. Aortic angiography and IVUS examination were performed using a designated catheter. After morphological evaluation, the pigs were subjected to median laparotomy, and both implanted grafts were excised with anastomosed native iliac arteries and the surrounding tissues. The explanted grafts were immediately rinsed with PBS, opened longitudinally, and examined macroscopically to evaluate luminal structures.

### Histology and immunostaining

The harvested grafts were briefly rinsed with PBS, fixed in 10% neutral-buffered formalin, and subsequently embedded in paraffin. Each specimen was sectioned longitudinally and circumferentially into 5 µm thickness. H&E staining was performed for the standard histological assessment of general vascular morphology. Additionally, immunofluorescence staining was conducted on paraffin-embedded sections of endothelial and smooth muscle cells. Briefly, the sections were deparaffinized, rehydrated, and blocked. The sections were then incubated with primary antibodies against desmin (monoclonal anti-DES LS-C352127; LSBio Inc., Seattle, United States) for smooth muscle cells (SMC) and CD31 (polyclonal anti-CD31, ab28364; Abcam, Cambridge, United States) for endothelial cells (EC), dehydrated, and mounted. All histological evaluations were performed by a board-certified pathologist and the analyzes were not conducted in a blinded manner.

### Statistical analysis

All data were expressed as mean ± standard deviation. Given the pilot nature of this study, statistical analyzes were considered exploratory. Unpaired t-test was performed to assess trends between groups, and effect sizes were calculated using Cohen's d. A *P*-value of 0.05, acknowledging limited statistical power due to the small sample size, was considered for trend identification. Statistical analyzes were performed using SPSS software (version 23.0; IBM, Armonk, NY, USA).

## Results

### Efficacy of decellularization

**Figure 1.**
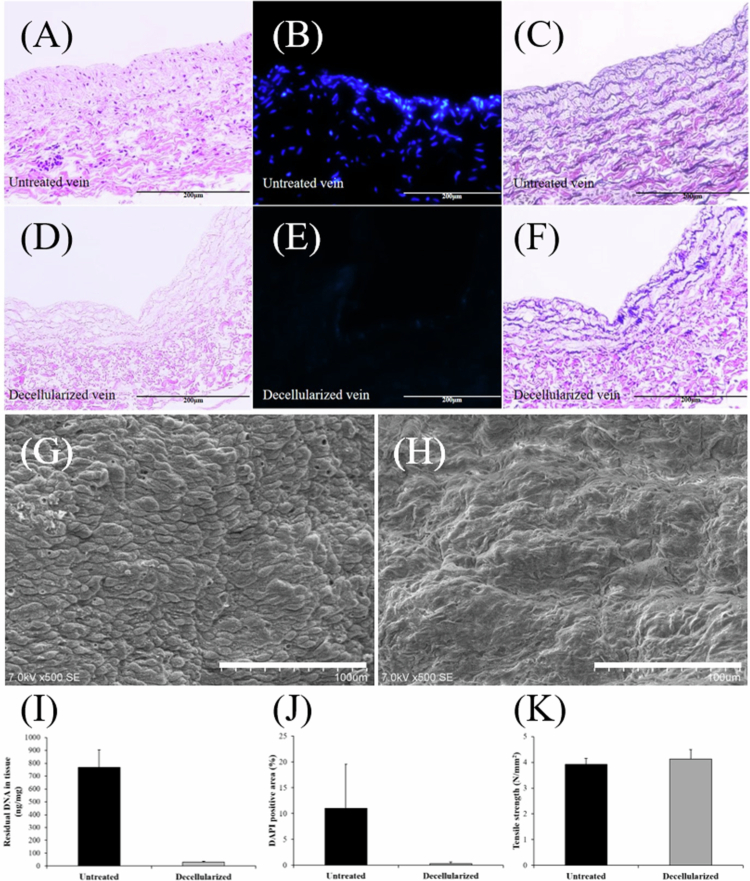
Evaluation of decellularization efficacy and scaffold mechanical properties. Representative histological, fluorescence, and ultrastructural images of native and decellularized porcine veins. H&E and DAPI staining show the removal of cellular components (A–B, D–E), and EVG staining confirms preservation of collagen and elastic fibers (C, F). SEM demonstrates removal of the endothelial layer (G: native, H: decellularized). Quantitative analyzes reveal marked reductions in residual DNA and DAPI-positive areas after decellularization, confirming effective cell removal while preserving extracellular matrix structure (I–J, *n* = 6 and 4 per group, respectively). Tensile testing showed no significant difference in ultimate tensile strength between native and decellularized scaffolds (K, *n* = 6 per group), indicating preserved mechanical integrity. Scale bars: 200 µm (A–F), 100 µm (G–H). SEM, scanning electron microscopy; H&E, hematoxylin and eosin; DAPI, 4′,6-diamidino-2-phenylindole; EVG, Elastica Van Gieson.

The detergent-enzymatic decellularization protocol using a perfusion system is a valid approach for removing host cells from porcine jugular veins without structural deterioration. Following decellularization, the absence of DNA, such as nuclei, was demonstrated by H&E and 4',6-diamidino-2-phenylindole staining (Figure 1A, 1B, 1D, and 1E). EVG staining revealed preservation of collagen and elastic fibers; both ECM components were necessary to maintain vessel intensity (Figure 1C, 1F). In native veins, a cobblestone-like appearance of the EC layer was visible on the intimal surface, whereas SEM observations of the decellularized scaffolds revealed a smoother intimal surface with successful removal of the EC layer (Figure 1G, 1H).

Spectrophotometric analysis demonstrated a marked reduction in residual DNA in the decellularized group compared with untreated controls (29.8 ± 7.0 vs. 768.2 ± 135.4 µg/mg dry tissue; *p* < 0.001; Cohen’s d = 7.70; *n* = 6 per group) (Figure 1I).

Quantitative analysis of DAPI-positive areas demonstrated a substantial reduction in nuclear material after decellularization (decellularized: 0.28 ± 0.19%, untreated: 11.0 ± 5.2%; *p* = 0.044; Cohen’s *d* = 1.77; *n* = 4 per group) (Figure 1J). Although the sample size was small, the marked reduction together with the histological findings confirmed that the decellularization protocol effectively removed cellular components while preserving the extracellular matrix.

### Mechanical properties

The ultimate tensile strengths of each tested group are shown in Figure 1K. The maximum average values for native and decellularized scaffolds were 3.92 ± 0.83 N/mm^2^ and 4.13 ± 0.51 N/mm^2^, respectively, with no significant differences between the groups (*p* = 0.40; Cohen’s d = 0.29; *n* = 6 per group). Thus, the mechanical characteristics of the decellularized scaffolds were considered tolerable for graft implantation in the in vivo trials.

### Implantation of TESG in porcine models

**Figure 2.**
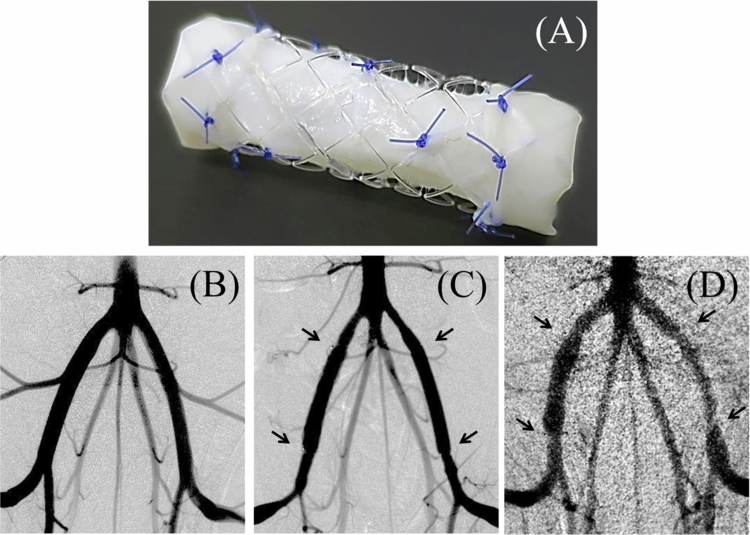
Construction of the TESG and angiographic evaluation in porcine models. The tissue-engineered stent graft (TESG) was constructed by suturing a decellularized porcine vein within a bioresorbable stent (BRS) using 6-0 absorbable sutures (A). The TESG was anastomosed to the right external iliac artery, and the control ePTFE graft was implanted on the left side in each animal. Digital subtraction angiography (DSA) performed before implantation (B), immediately after implantation (C), and on postoperative day 14 (D) showed maintained luminal patency and morphological preservation of the TESG throughout the study period. Arrows indicate anastomotic sites (*n* = 3). ePTFE, expanded polytetrafluoroethylene.

Prior to the utilization of the TESG in endovascular treatment, surgical graft implantation was performed to assess short-term graft patency and hemodynamic intensity in juvenile porcine models. As described previously, the decellularized porcine jugular vein and BRS were sutured with absorbable sutures to create a TESG (Figure 2A). Porcine external iliac arteries were replaced with either a TESG or an ePTFE PG using end-to-end anastomosis (*n* = 3 each). The TESG consistently implanted in the right external iliac artery and the PG in the left side of each animal. All surgical procedures were performed without complications or perioperative mortality. The technical success rate was 100% in all implant cases.

### Hemodynamic evaluation of implanted grafts

**Figure 3.**
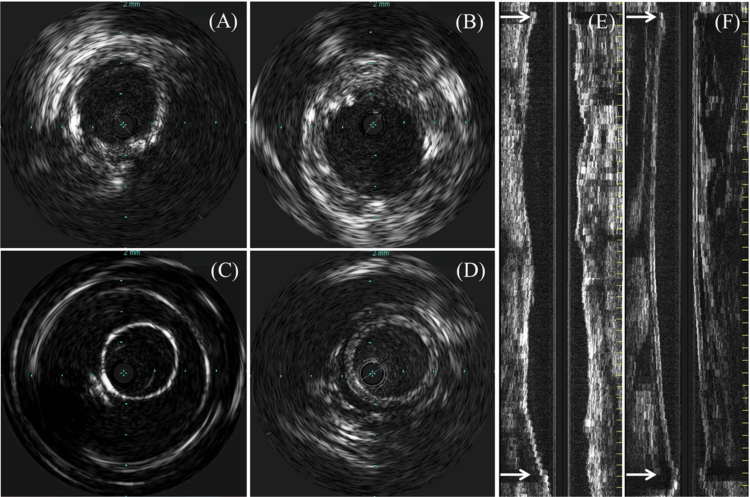
IVUS evaluation of implanted grafts. Intravascular ultrasound (IVUS) images in cross-sectional and longitudinal views on days 0 (A, C) and 14 (B, D–F). TESG (A, B, and E) preserved luminal architecture, whereas ePTFE grafts (C, D, and F) showed mild luminal thickening and stenosis at the distal anastomosis. Arrows indicate anastomotic sites.

Intraoperative angiography (Figure 2B-D) and IVUS (Figure 3A-F) were performed to determine the accuracy of the anastomotic procedures and hemodynamic behavior of the implanted grafts. Six grafts were successfully anastomosed to porcine iliac arteries. Intraoperative graft patency was confirmed in all animals and anastomotic stenosis was not evident (Figure 2C). On day 14, all the TESGs maintained patency, showing sufficient peripheral perfusion without any structural deterioration on angiography (Figure 2D). However, of the three PGs, only one graft was clearly patent; the other grafts showed one anastomotic stenosis and one graft occlusion. In the IVUS analysis, the TESGs maintained their architectural behavior, showing no evidence of aneurysm formation or devoting rupture.

### Histological examination of explanted grafts

**Figure 4.**
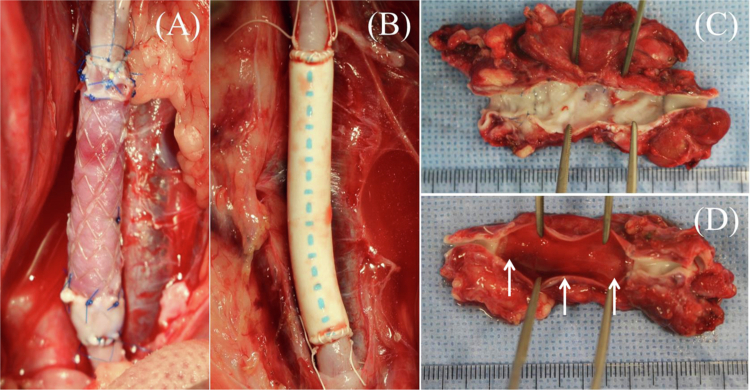
Macroscopic views of implanted and explanted grafts. Each graft was anastomosed to the iliac artery with an end-to-end anastomosis: (A) TESG, (B) ePTFE graft. TESG (A, C) and ePTFE grafts (B, D) were explanted on day 14. TESG exhibited smooth luminal surfaces, whereas ePTFE grafts showed fibrin deposition and luminal stenosis with mild thrombus formation.

**Figure 5.**
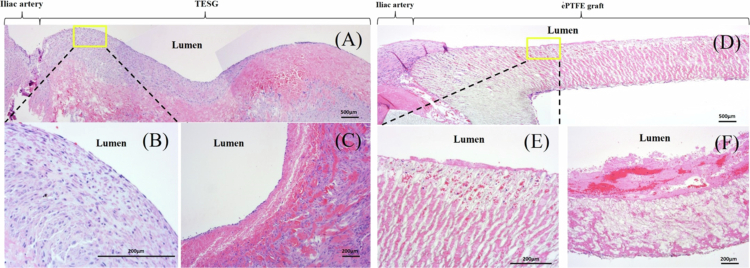
Representative hematoxylin and eosin (H&E) histological evaluation of each graft at 2 weeks after implantation. In the TESG group (A, B, and C), H&E staining showed the transluminal repopulation of endothelial and SMC-like cells in the graft wall. In the PG group (D, E, and F), platelets adhered to luminal surface, and few repopulated cells were detected through the graft. (A, D) longitudinal section, (B, E) magnified view of the peri-anastomotic region, (C, F) circumference section of B.

All grafts were harvested 14 days after surgical implantation (Figure 4A-D). Macroscopically, the TESG exhibited a smooth luminal surface without thrombus adhesion (Figure 4C). In contrast, a fibrin layer formed along the PG lumen with slight luminal stenosis (Figure 4D). H&E staining was performed to analyze the general morphological characteristics of the explanted grafts (Figure 5A-F). In the TESG group, host cell repopulation in the acellular decellularized scaffold was detected from the anastomotic sites to the mid-graft on day 14 (Figure 5A). A monolayer of flattened cells, similar to EC, consecutively covered the luminal surface (Figure 5B, 5C). Multiple spindle cells, morphologically similar to SMC or myofibroblasts, were also observed beneath the EC-like monolayer. However, in the PG group, the fibrin thrombus adhered to the luminal surface, and infiltration of EC-like or SMC-like cells was undetectable (Figure 5D-F).

**Figure 6.**
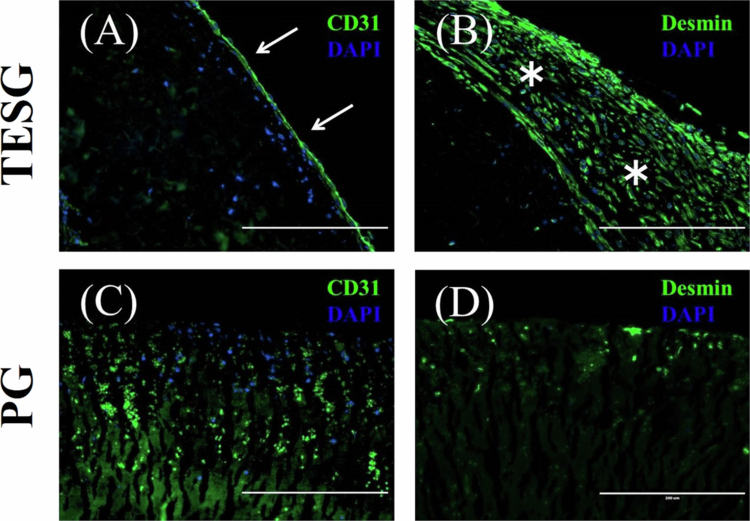
Representative immunofluorescence staining of implanted grafts in longitudinal sections. In TESG group (A, B), CD31( + ) endothelial cells (green; arrows in A) and desmin( + ) smooth muscle cells (green; asterisks in B) successively repopulated from native iliac artery to TESG. However, in PG group (C, D), the absence of any host cells from the anastomosis was confirmed. (Scale bars: 200 μm).

Immunofluorescence staining further confirmed these findings. In the TESG group, CD31-positive cells formed a continuous monolayer from the anastomosis, showing repopulation of ECs in the implanted stent graft (Figure 6A). SMC-like cells under the endothelial layer were visualized in H&E staining and further examined by anti-desmin immunostaining. These cells specifically and densely stained positive for desmin, suggesting the recellularization of SMCs into decellularized scaffolds (Figure 6B). In contrast, CD31 and desmin expression were not evident in the PG group, indicating poor host cell infiltration from the native artery (Figure 6C, 6D).

## Discussion

The present study aimed to develop a novel TESG, expected potential for eventual integration with autologous tissue by combining a decellularized graft with a BRS. Histopathological evaluation confirmed the efficacy of the decellularization protocol, as evidenced by the disappearance of nuclei and flattening of the luminal surface structure of the decellularized porcine vessels. The mechanical properties of the TESG created through decellularization are presumed to possess sufficient endurance for subsequent in vivo implantation.

The primary objective of decellularization is to eliminate all cellular components while preserving the native histoarchitecture of the ECM. This process is critical for reducing the antigenicity of allografts, thereby minimizing the immune responses after implantation. In the present study, the decellularization protocol effectively lowered residual DNA content while maintaining the structural integrity of the vascular ECM. Histological analysis confirmed the absence of cellular elements and quantitative assessment demonstrated a significant reduction in DNA content, validating the efficacy of the decellularization process.

Angiography and IVUS examinations in the porcine model displayed acceptable graft patency and morphological preservation of the TESG during short-term follow-up compared to the PG. In the clinical setting, degeneration of the TESG component may lead to aneurysm formation or vessel rupture with subsequent life-threatening graft failure. Therefore, these results are significant because the TESG had sufficient mechanical properties to tolerate hemodynamic stress, even for a short period. Good preservation of the ECM even after decellularization presumably contributed to maintaining the morphological structure of the TESG.

The mechanical properties of artificial grafts have been proven to be critical for preserving their long-term patency. Compliance mismatch of elasticity between the grafts and native vessels has previously been proposed as one of the main causes of graft failure due to endothelial hyperplasia.^20^ The occurrence of a compliance mismatch may result in graft wall thickening or expansion and the formation of graft occlusions or aneurysms, ultimately leading to implant failure. In our porcine model, TESG demonstrated favorable patency compared to PG, which is likely attributable to better compliance matching with recipient native vessels. This suggests that the decellularized scaffold possessed a mechanical behavior that was more physiologically aligned with the native arteries.

Although the implantation studies were conducted for a short period, signs of host cell recruitment from the perianastomotic regions to the acellular scaffold were observed. As demonstrated by the histological findings, EC- and desmin-positive SMC-like host cells were dense near the perianastomotic regions, presumably migrating from the adjacent anastomosed arteries; unfortunately, less visible in the mid-graft section. Given that the follow-up period in this study was limited to 14 days, long-term remodeling processes, including complete endothelialization, scaffold degradation, and vessel maturation, could not be fully assessed. Future investigations with longer observation periods are required to confirm the durability and biological integration of the TESG.

ECs play a critical role in the success of decellularized vascular grafts by contributing to thrombo-resistance, regulating vascular tone, and maintaining long-term patency. Previous reports have revealed that the presence of a confluent monolayer of ECs is promising for decreasing the thrombogenicity of the graft and prolonging patency.^7^ A functional endothelial layer not only serves as a barrier against thrombogenic stimuli, but also modulates inflammation and SMC proliferation through the release of vasoactive mediators. In decellularized vascular grafts, early recellularization with ECs is expected to restore the physiological vascular function and ultimately promote seamless integration with the host tissue.

Remedy is a BRS used to create a TESG that is innovative for the treatment of vascular diseases and stenotic lesions. PLLA, the main component of the BRS, undergoes gradual hydrolytic degradation with subsequent metabolism through the Krebs cycle, resulting in complete resorption within approximately 12-18 months in vivo.^16^ This degradation period is expected to provide adequate mechanical support during the early healing phase and enable progressive biointegration of the TESG thereafter. Their primary advantages include obviating the need for future re-intervention, post-stent absorption, and promoting long-term vascular restoration compared with traditional metallic stents. A recent prospective multicenter study by Obara et al. evaluated the long-term outcomes of the Remedy stent in patients with iliac artery disease and demonstrated a 5-year target lesion revascularization-free rate of 85.4% and favorable safety results without any device-related deaths or major amputations.^21^ The study confirmed sustained clinical and hemodynamic improvements, highlighting the potential utility of BRS in selected iliac lesions, especially in the common iliac artery. Although the current pilot study did not directly assess the scaffold function of the Remedy stent, these findings suggest that current bioresorbable scaffolds may offer a promising and safe alternative to modular metallic stents for the development of TESG.

Conventional BRSs have been developed and clinically evaluated primarily for stenotic lesions such as coronary artery stenosis and peripheral artery disease; however, their application as components in the treatment of arterial aneurysms has not been previously reported. Therefore, this concept represents a novel and promising direction for vascular therapy.

Takeuchi et al. demonstrated the successful integration of a partially degradable TESG combined with a synthetic TEVG and a metallic stent in a porcine aortic model.^15^ Compared with synthetic polymer-based TEVGs, decellularized TEVGs offer several distinct biological advantages. Although polymer-based grafts can induce host tissue ingrowth through controlled biodegradation and inflammatory signaling, they lack the complex ultrastructure and biochemical cues inherent to native vascular tissue. In contrast, decellularized vessels preserve the native ECM architecture, including collagen, elastin, and basement membrane components, which are critical for supporting host cell adhesion, migration, and endothelialization.^22^ Moreover, decellularized TEVGs exhibit superior biomechanical compliance and reduced thrombogenicity, facilitating rapid integration and minimizing adverse host responses.^13^ These properties suggest that decellularized TEVGs may provide a more physiologically relevant and immunologically inert alternative for SG applications. Unlike metallic stents, which remain permanently in the body and may interfere with long-term remodeling, our TESG employs a BRS that degrades over time, with residual autologously integrated tissues. This feature offers a conceptual advantage by promoting complete vascular restoration and eliminating long-term retention of foreign materials.

This study has few limitations that warrant consideration. First, although the TESG demonstrated acceptable short-term patency and mechanical performance, its long-term durability and functional integrity remain uncertain. Extended observation periods are necessary to assess the stability of the graft and the risk of late complications, such as stenosis, migration, or structural degradation. Second, although TESG was designed with the expectation of progressive bioresorption and host tissue integration, these dynamic morphological and biological changes could not be adequately evaluated within the short follow-up period of the present study. Third, the translational relevance of this study to future clinical endovascular applications remains uncertain because the implantation approach involves direct suturing of the SG to native vessels. This fundamentally differs from intraluminal deployment in endovascular procedures, where the graft is secured by radial force and friction. These differences in the implantation mechanics may significantly influence host cell repopulation, fixation strength, and degradation kinetics.

Looking ahead, several key aspects need to be refined to advance the clinical translation of TESG technology. These include improving sterilization and storage methods to preserve extracellular matrix bioactivity and addressing regulatory challenges related to manufacturing standardization and biosafety evaluation. Encouragingly, recent clinical use of decellularized vascular grafts has demonstrated the feasibility and safety of xenogeneic-derived scaffolds in humans, indicating that decellularized vascular grafts implantation is clinically acceptable.^23^ Moreover, xenogeneic sources provide abundant graft material with a wide range of vessel diameters, facilitating adaptation to diverse human vascular anatomies. These established clinical experiences form a solid foundation for extending the TESG concept to endovascular applications.

Further research focusing on long-term in vivo evaluation, optimization of the TESG platform for endovascular delivery, and validation of its mechanical stability and biological performance in large-animal models will help advance this technology toward clinical application. Collectively, these efforts are expected to support the development of next-generation bioresorbable stent grafts capable of achieving complete vascular restoration.

## Conclusion

A TESG composed of a porcine-derived decellularized scaffold and a BRS made of PLLA demonstrated favorable biological responses both in vitro and in vivo. Following implantation, essential host cells responsible for maintaining graft integrity and function were successfully repopulated from the surrounding native tissues, indicating a high degree of biocompatibility and integration. To the best of our knowledge, this study is the first to develop and evaluate a biologically interactive and resorbable SG designed for endovascular applications. These findings offer potential advances in the development of next-generation endovascular devices. With further engineering refinements tailored for endovascular deployment, this TESG platform may serve as a next-generation medical device capable of mitigating the risks associated with conventional SGs, such as migration, infection, and long-term foreign body reactions. However, further preclinical studies and long-term evaluations are warranted to validate the safety, durability, and functional performance of this novel TESG before clinical translation.

## Data Availability

No datasets were generated or analyzed during the current study. All data supporting the findings of this study are available within the article.
